# Acute Myocardial Infarction in Patients with Hereditary Thrombophilia—A Focus on Factor V Leiden and Prothrombin G20210A

**DOI:** 10.3390/life13061371

**Published:** 2023-06-12

**Authors:** Minerva Codruta Badescu, Lăcrămioara Ionela Butnariu, Alexandru Dan Costache, Liliana Gheorghe, Petronela Nicoleta Seritean Isac, Adriana Chetran, Sabina Andreea Leancă, Irina Afrăsânie, Ștefania-Teodora Duca, Eusebiu Vlad Gorduza, Irina Iuliana Costache, Ciprian Rezus

**Affiliations:** 1 Department of Internal Medicine, “Grigore T. Popa” University of Medicine and Pharmacy, 700115 Iasi, Romania; minerva.badescu@umfiasi.ro (M.C.B.); isac_petronela@yahoo.com (P.N.S.I.); adriana.ion@umfiasi.ro (A.C.); sabinaandreea-leanca@email.umfiasi.ro (S.A.L.); irina-demsa@email.umfiasi.ro (I.A.); stefania-teodora.duca@email.umfiasi.ro (Ș.-T.D.); irina.costache@umfiasi.ro (I.I.C.); ciprian.rezus@umfiasi.ro (C.R.); 2 III Internal Medicine Clinic, “St. Spiridon” County Emergency Clinical Hospital, 700111 Iasi, Romania; 3 Department of Mother and Child Medicine, “Grigore T. Popa” University of Medicine and Pharmacy, 700115 Iasi, Romania; eusebiu.gorduza@umfiasi.ro; 4 Cardiovascular Rehabilitation Clinic, Clinical Rehabilitation Hospital, 700661 Iasi, Romania; 5 Department of Radiology, “Grigore T. Popa” University of Medicine and Pharmacy, 700115 Iasi, Romania; 6 Radiology Clinic “St. Spiridon” County Emergency Clinical Hospital, 700111 Iasi, Romania; 7 Cardiology Clinic, “St. Spiridon” County Emergency Clinical Hospital, 700111 Iasi, Romania

**Keywords:** myocardial infarction, factor V Leiden, prothrombin G20210A, thrombophilia

## Abstract

Factor V (FV) Leiden and prothrombin G20210A are the most common hereditary thrombophilias. While their role in venous thromboembolism is well known, there are still uncertainties regarding their relationship with arterial thrombotic events, especially coronary ones. Our research, based on an in-depth analysis of the available literature, provides up-to-date information on the relationship between FV Leiden and prothrombin G20210A and acute myocardial infarction. FV Leiden and prothrombin G20210A screening should be implemented only in select cases, such as acute coronary syndrome in young individuals and/or in the absence of traditional cardiovascular risk factors and/or in the absence of significant coronary artery stenosis at angiography. Their identification should be followed by the implementation of optimal control of modifiable traditional cardiovascular risk factors to reduce the risk of recurrent events and genotyping and genetic counseling of all family members of affected cases for proper prophylaxis. An extended dual antiplatelet therapy (DAPT) may be considered, given the lower risk of bleeding under DAPT conferred by FV Leiden.

## 1. Introduction

Thrombophilia can be hereditary or acquired. In the presence of a genetic substrate, the prothrombotic state can result from increased concentrations of procoagulant factors (gain-of-function disorders) or deficiencies/dysfunctions in the endogenous anticoagulant system (loss-of-function disorders). The gain-of-function disorders mainly include the presence of factor V (FV) Leiden and/or the G20210A mutation in the prothrombin gene. Loss-of-function disorders mainly involve proteins C and S and antithrombin III (AT III) [1]. Loss-of-function disorders are less common than gain-of-function disorders, but they are much more powerful risk factors for thrombosis.

The relationship between hereditary thrombophilia and venous thromboembolism (VTE) has solid evidence. The risk for a first VTE is increased 5 to 7 times in individuals heterozygous for FV Leiden and 25 to 50 times in those homozygous for this mutation [2]. Furthermore, heterozygosity for FV Leiden is associated with a 42% increase in the risk of VTE recurrence [3]. Heterozygosity for prothrombin G20210A is associated with a 3 to 4 times increase in the risk for a first VTE. Homozygosity for prothrombin G20210A is much rarer. However, its association with VTE has been reported [4]. Overall, FV Leiden and prothrombin G20210A explain 20–30% of cases of first VTE and 60–70% of cases of recurrent VTE and VTE in young people.

Based on these data, it was assumed that hereditary thrombophilia plays a role in arterial thrombosis as well, but this hypothesis was not always validated. While some studies found that FV Leiden increases the risk of ischemic stroke by 33–53% [5,6,7], mainly in young adults [8,9], others showed no association between FV Leiden and arterial thrombotic events [10,11]. Data regarding prothrombin G20210A in stroke patients were inconsistent, as well. One study reported a 1.5 times increased risk of ischemic stroke in prothrombin G20210A carriers for all ages and 1.8 times increased risk when only children and young adults were included in the analysis [12]. Other studies showed no association between prothrombin G20210A and the risk of stroke [7,13].

Studies published more than 20 years ago showed that FV Leiden and prothrombin G20210A increase the risk of myocardial infarction (MI) by 40% and 50%, respectively [14]. The contribution of thrombophilia seemed to be more important at young ages when the atherosclerotic load of coronary arteries is low. In patients under the age of 50, survivors of a first MI, FV Leiden, and prothrombin G20210A were 4.7 and 4.4 times, respectively, more prevalent in those who had coronary artery stenosis <50% compared to those who had at least one coronary artery stenosis ≥50% [15]. However, other studies have shown that neither FV Leiden nor prothrombin G20210A increases the risk of MI [11,13,16]. A family-based study investigating genetic factors associated with a high frequency of premature-onset acute coronary syndromes (ACS) found only a statistically marginal influence of FV Leiden [17]. In a Danish cohort, the risk of MI in heterozygotes and homozygotes FV Leiden carriers was similar to that of the general population [18].

In 2006, Ye et al. [19] published the results of a large meta-analysis of 191 studies on the relevance of polymorphisms of several hemostatic genes to coronary artery disease (CAD), including FV Leiden and prothrombin G20210A. They provided the first clear indication of the existence of a moderate and highly significant increase in per-allele relative risk for MI in FV Leiden and/or prothrombin G20210A carriers. This risk was 1.22 (95% CI 1.10–1.35) for FV Leiden carriers and 1.25 (95% CI 1.05–1.50) for prothrombin G20210A carriers [19]. A 30% increase in the risk of CAD in carriers of one of the two thrombophilic traits compared to non-carriers was considered significant.

In the last two decades, the primary and secondary prevention measures of CAD have improved significantly; therefore, it is important to know if individuals with FV Leiden and/or prothrombin G20210A—as the most common thrombophilic traits in the general population—currently require special management. Our in-depth analysis of the recent literature aims to provide up-to-date information on some practical aspects, namely: which individuals would benefit from screening for these thrombophilic traits; if antithrombotic therapy should be customized for MI survivors according to these thrombophilic traits; and what the risk of recurrence of coronary events in patients with these thrombophilic traits is. In order to fulfill this goal, we conducted an extensive literature search and only included original studies published in English in the analysis. As a starting point, we took the excellent meta-analysis by Ye et al. based on studies published before January 2005 [19].

## 2. Factor V Leiden

**Figure 1:**
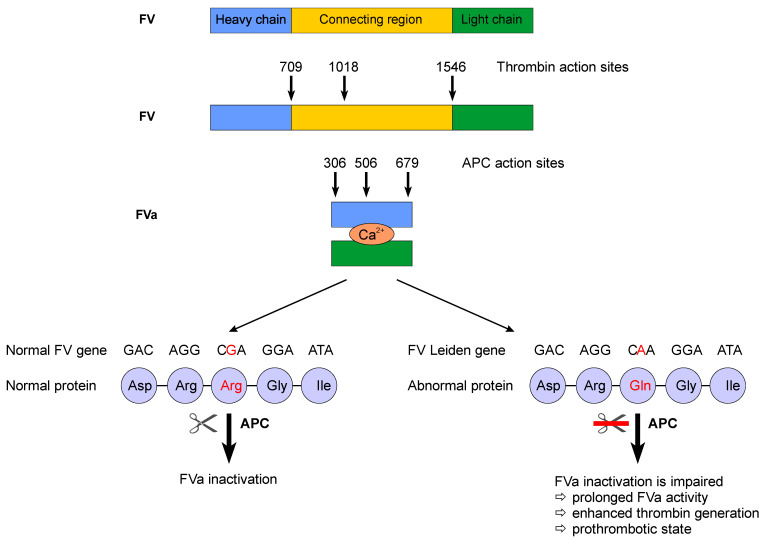
Factor V Leiden is an abnormal protein, the result of a single point mutation at position 1691G/A of the FV gene. Its inactivation is impaired, which leads to a prothrombotic state.

Factor V plays an important role in coagulation. In its activated form (FVa), it binds to activated factor X (FXa) and forms the prothrombinase complex at the surface of activated platelets. This event is followed by the generation of a large amount of thrombin and, subsequently, by the formation of fibrin [20]. One of the counter-regulatory mechanisms is the protein C pathway. Activated protein C (APC) coupled with its cofactor—protein S—inactivates FVa, thereby exerting inhibitory effects on coagulation. Moreover, FV and protein S act synergistically with APC in the inactivation of activated factor VIII (FVIIIa) assembled in the tenase complex [21]. A single-point mutation in the position 1691G/A of the FV gene results in the synthesis of an abnormal protein (FV Leiden). Because the mutation involves one of the three sites of action of APC, inactivation of FVa will be slower, and inactivation of FVIIIa will be impaired, resulting in a prothrombotic state. FV Leiden is the most common cause of resistance to APC (Figure 1).

Factor V Leiden—the most common hereditary thrombophilia—has an unequal worldwide distribution between ethnic groups. Five percent of Caucasians are heterozygous for this mutation, and less than 1% are homozygous. In the Western population, the reported prevalence is 3–7% [22]. In Greece and Cyprus, the prevalence is double, at 14% and 12.2%, respectively. Studies from Turkey, Azerbaijan, and Egypt also report an increased prevalence of this mutation in the general population—a prevalence of 7.1–10.3%, 14%, and 18.5%, respectively [23,24]. FV Leiden is sporadically found in other ethnic groups [2]. These data are important for interpreting the results of studies conducted on various populations across continents.

### 2.1. Myocardial Infarction in FV Leiden Carriers

In the elderly, atherothrombotic events underlie almost all ACS. Although FVa has a prothrombotic effect, its inactivation plays only a minor role in the thrombotic complications of atherosclerotic plaque; therefore, FV Leiden does not contribute to the occurrence of the first MI at advanced ages [25]. In elderly patients with angiographically documented advanced CAD, no correlation was found between the incidence of MI and the presence of FV Leiden [26]. Recent studies showed that the prevalence of FV Leiden in MI patients with a mean age of 69.5 years is not different between sexes [27]. Moreover, starting from the age of 55, the prevalence of FV Leiden is similar in MI patients and age-matched controls [28].

Unlike adults, young patients have a different profile of cardiovascular risk factors and the extent of atherosclerotic burden. Therefore, coronary atherosclerosis is less prevalent, and the contribution of other factors, such as thrombophilia, should be considered. Coronary angiographic evaluation of young patients with acute MI usually identified single-vessel disease, thereby showing that coronary atherosclerosis is not extensive. Of note, the left main is rarely affected [29]. Furthermore, the likelihood that a thrombophilic genetic factor has a significant role in the pathogenesis of acute MI can be better assessed in the young than in the elderly because, at a young age, the factors related to aging are weak confounders of the effect of the genetic component. 

Patients who develop MI at younger ages tend to have a higher prevalence of FV Leiden than the general population. A study that included only women aged 18 to 44 years without a history of CAD found that FV Leiden increased the risk of first acute MI by 2.4 times [30]. More importantly, this study highlighted the additive effect of traditional cardiovascular risk factors on MI incidence in FV Leiden carriers. The risk of MI was 32 times higher (95% CI 7.7–133) in carriers who smoked and 25 times higher (95% CI 4.5–194) in carriers with either, or a combination of, obesity, hypertension, diabetes, or hypercholesterolemia than in women without both FV Leiden and traditional cardiovascular risk factors.

Analysis of a large cohort of Italian patients who developed MI before the age of 45 showed that FV Leiden was 1.66 times more prevalent in patients with MI than in controls [31]. A trend towards a higher prevalence of FV Leiden was observed in patients without significant coronary artery stenosis. Surprisingly, FV Leiden did not increase the risk of acute MI in patients with normal cholesterol levels, whereas, in those with hypercholesterolemia, FV Leiden further increased the risk by two times. Thus, in patients with concomitant FV Leiden and hypercholesterolemia, the risk of acute MI was about 3.5 times higher than in patients without FV Leiden and with normal cholesterol levels. Although the controls were individually matched to the cases in terms of age, sex, educational level, and geographic origin, the results of this study must be interpreted within the limits of its case–control design.

There is evidence that the contribution of an inherited predisposition to thrombosis in the production of a MI is relatively weak in itself and weaker than that of traditional risk factors [32]. Still, when one or more of the classic cardiovascular risk factors, such as hypertension, diabetes mellitus, obesity, dyslipidemia, and smoking, overlap the inherited prothrombotic substrate, the risk of MI increases significantly [14,30].

An extremely high prevalence of FV Leiden was reported by an Egyptian study [24]. FV Leiden was identified in 52% of patients with MI, with a mean age of 52.2 years. Although a high prevalence of FV Leiden of 18.5% was reported in healthy controls—the expression of the prevalence variability with ethnicity—the prevalence of the mutation was almost fivefold higher in cases than in controls. The prevalence of heterozygosity and homozygosity for FV Leiden was over fourfold and over eightfold higher, respectively, in patients with MI than in controls.

Another aspect of interest is the type of MI that FV Leiden carriers develop and the magnitude of myocardial necrosis. A recent meta-analysis showed that the presence of FV Leiden did not influence either the type of ACS—ST-segment elevation myocardial infarction (STEMI) vs. non–ST-segment elevation ACS—or the extent of myocardial necrosis [33]. In the era of rapidly available coronary angiography in patients with ACS, the diagnosis of myocardial infarction with nonobstructive coronary arteries (MINOCA) is established in up to 14% of cases [34]. MINOCA is diagnosed when there is clinical evidence of MI, and no epicardial coronary has a stenosis ≥50% on angiography. Among the major etiological substrates of MINOCA, thrombophilia ranks third after structural myocardial dysfunction (cardiomyopathies) and coronary spasm. Atherosclerotic plaque rupture or erosion is followed by the formation of a thrombus on its surface. MI occurs via distal embolization or transient complete thrombosis with spontaneous thrombolysis [35]. Patients with MINOCA tend to be younger than those with obstructive CAD, with an average age of 55 years and with a lower prevalence of dyslipidemia.

The incidence of congenital and acquired hypercoagulability states is higher in patients with MINOCA than in the general population. Among 84 MINOCA patients, FV Leiden was found in 14.3% of patients [36]. Congenital coagulation disorders were identified in 23.8% of MINOCA patients [37]. Da Costa et al. reported that of 91 patients with acute MI and normal coronary arteries on angiography, 12,8% had congenital coagulation disorders, mainly a resistance to APC [38]. Another study reported that the prevalence of resistance to APC in MI patients with normal coronary angiogram was 9.1%, while in MI patients with stenosed or occluded arteries, this prevalence was 3.8%, equal to that of age- and sex-matched healthy controls [39]. In newer studies, the most common thrombophilia trait is FV Leiden, found in 10% and up to 14.3% of MINOCA patients [36,40].

The systematic review that provided the first comprehensive overview of MINOCA patients identified eight studies that investigated the presence of inherited thrombotic disorders in this particular category of MI patients [34]. Of the 378 patients who were screened for thrombophilia, 14% had an inherited thrombotic disorder. APC resistance/FV Leiden was present in 12% of 344 patients. This prevalence is higher than that of FV Leiden in the Western population, which is 3–7% [22]. A higher prevalence of FV Leiden was found in patients with MINOCA compared to patients with MI and obstructive coronary artery stenosis in several studies (12.1% vs. 4.5% [41]; 11.7% vs. 4.3% [15], respectively). When only patients under the age of 50 were analyzed, the prevalence of FV Leiden was 4.7 times higher in MINOCA patients than in controls (14.6% vs. 3.6%) [15]. In FV Leiden carriers, MINOCA expresses more as non-ST segment elevation MI (NSTEMI) than STEMI (18.2% vs. 10%).

### 2.2. The Risk of Recurrence of MI

There is much evidence that FV Leiden is a strong contributor to VTE recurrence [3]. It is also associated with accelerated atherosclerosis and early restenosis after revascularization in patients with femoral-popliteal angioplasty with artificial grafts [42].

However, currently available data do not support the role of FV Leiden in increasing the risk of recurrence of coronary events. In a study with a mean follow-up period of 11 years, 47% of patients with a history of MI experienced recurrent events, such as non-fatal MI, unstable angina, revascularization, or death from CAD, but no correlation could be established between the presence of FV Leiden and the risk of recurrence of fatal or non-fatal ischemic coronary events [43].

These results are reinforced by a recent large-scale individual-level data meta-analysis. Based on the analysis of 25 studies assessing the role of FV Leiden in patients at high risk of atherothrombotic events, no association was found between FV Leiden and subsequent or recurrent atherothrombotic events [44]. Furthermore, neither traditional cardiovascular risk factors, including hypertension, overweight/obesity, and diabetes, nor treatment with statins or antiplatelet agents caused any change in the lack of association between FV Leiden and the risk of recurrent MI or death from CAD in patients with established and treated CAD.

In MINOCA patients, the risk of future vascular thrombotic events (venous or arterial) is three times higher in those with inherited coagulation abnormalities than in those without any [37]. Although the presence of inherited coagulation disorders is an independent predictor of future thrombotic events, reinfarction occurred in only 2 of 12 patients with MINOCA and congenital thrombophilia.

Considering the available data, it is justified to conclude that once the diagnosis of CAD is established and the disease is treated, no additional benefit is obtained from the identification of FV Leiden [45]. However, new rationales for identifying the presence of FV Leiden after an ACS are emerging.

Firstly, unexpected data come from PLATO and CURE trials, which exclusively enrolled patients with ACS [46,47]. The composite outcome of death from cardiovascular causes, non-fatal MI, or stroke was significantly reduced in the PLATO trial by the aspirin plus ticagrelor regimen compared to the aspirin plus clopidogrel regimen and in the CURE trial by the clopidogrel plus aspirin regimen compared to the aspirin alone regimen, the magnitude of the effect being greater in FV Leiden carriers. A post hoc analysis found a statistically non-significant higher protective benefit in FV Leiden carriers for dual antiplatelet regimen (HR, 0.67 [95% CI, 0.50–0.92]) compared to the single antiplatelet regimen (HR, 0.97 [95% CI, 0.50–1.89]) [44]. The FV fraction contained in platelet granules could explain this phenomenon. It represents about 20% of all FV; it is released only upon platelet activation and has a stronger hemostatic effect than circulating FV [48].

Secondly, the first data on the impact of FV Leiden on bleeding risk in patients with ACS using antiplatelet therapy was recently published [49]. It highlights the protective effect of FV Leiden on bleeding complications in patients under dual antiplatelet therapy (DAPT). A meta-analysis of ACS patients from three trials, namely CURE, PLATO, and PopGen, showed that FV Leiden carriers have a lower risk of bleeding under antiplatelet treatment than non-carriers. Of note, patients were mainly on DAPT. FV Leiden carriers had a lower incidence of the composite outcome consisting of major or minor bleeding than non-carriers. The difference was determined by the minor bleeding and not by the major ones. The association of FV Leiden with bleeding was the same regardless of the P2Y12 receptor inhibitor used. However, FV Leiden was associated with less bleeding in patients treated with single antiplatelet therapy than with DAPT. Furthermore, this analysis highlighted that under antiplatelet treatment, the FV Leiden was not associated with an increased incidence of atherothrombotic events. Thus, the lower risk of bleeding under DAPT conferred by factor V Leiden was not counterbalanced by a higher risk of ischemic events.

## 3. Prothrombin G20210A

**Figure 2:**
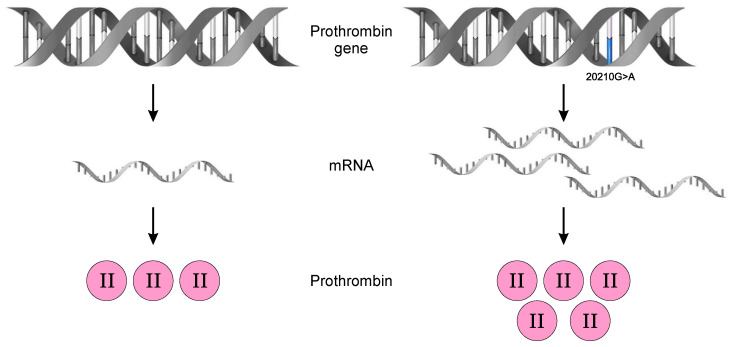
The 20210G>A mutation in the prothrombin gene determines the increase in mRNA production and implicitly in the amount of prothrombin. Prothrombin function is normal.

Prothrombin is the precursor of thrombin, a serine protease with the ability to exercise both procoagulant and anticoagulant functions. Depending on the binding with Na^+^, thrombin has two forms: Na^+^-free slow form and Na^+^-bound fast form. When bound to Na^+^, thrombin is highly active and performs procoagulant functions, such as the conversion of fibrinogen to fibrin; activation of plasma coagulation factors FV, FVIII, FXI, and FXIII; activation of platelets; and inhibition of fibrinolysis by activating thrombin activatable fibrinolysis inhibitor (TAFI) and inactivating ADAMTS13. When free of Na^+^, thrombin exerts an anticoagulant effect with the assistance of thrombomodulin. The affinity of thrombomodulin to thrombin is much higher than the affinity of thrombin to fibrin. Therefore, when thrombin binds to thrombomodulin, the thrombin activity is switched from a procoagulant to an anticoagulant function. The thrombin-thrombomodulin complex activates the protein C pathway, resulting in the inactivation of FVa and FVIIIa. Furthermore, the binding of thrombin to antithrombin leads to thrombin inactivation [50]. The G20210A allele is associated with increased production and plasma levels of prothrombin, increasing the endogenous thrombin potential (Figure 2). Prothrombin production is increased by approximately 30% in heterozygotes and 70% in homozygotes [13]. Thus, both excessive activation of the coagulation cascade and attenuation of fibrinolysis participate in the prothrombotic state.

Prothrombin G20210A is the second most common hereditary thrombophilia, but its geographical distribution is uneven [51]. In Europe, the prevalence is higher in the countries from the south than in the north of the continent, at 3% and 1.7%, respectively. In Asia, there is a big difference between Middle East populations, which have a prevalence of 2.5% to as high as 12.25%, and the rest of the continent, where the mutation is absent. In Africa, the prevalence ranged between 0 and 3.9%, with the highest values in the northern countries closer to Europe. A prevalence of 2.4 to 4.1% was reported in Australia and 2–2.5% in South America. In the US, the prevalence was 0–5%, the mutation affecting mainly Caucasians and Hispanics.

### 3.1. Myocardial Infarction in Prothrombin G20210A Carriers

Efforts to unravel the correlation between prothrombin G20210A and the risk of MI have ended in conflicting reports that have made it impossible to draw a consistent conclusion over time. One study reported that in the elderly, the prevalence of prothrombin G20210A was four times higher in MI patients than in age-matched controls [28]. Two systematic reviews conducted in the early 2000s found that prothrombin G20210A did not increase the risk of MI in the general population of all ages but only in individuals younger than 55 years. In them, the risk of MI was increased approximately 1.8 times [52,53]. A Moroccan study showed that the prevalence of prothrombin G20210A in patients with MI was 2.4 times higher in young patients compared to the elderly [54]. The most recent meta-analysis, conducted on 34 studies, demonstrated that prothrombin G20210A was associated with an increased risk of MI only in Caucasians younger than 55 years and not in other ethnic groups or individuals older than 55 years [55]. Apparently, the prevalence of prothrombin G20210A does not differ between sexes in elderly patients with MI [27].

It becomes clear that similar to what has been observed in patients with FV Leiden, the role of prothrombin G20210A in the occurrence of ACS appears to be more significant in patients with reduced rather than extensive coronary atherosclerosis. The prevalence of this thrombophilic trait was double among patients with no or one vessel disease (4.4%) than in those with multi-vessel disease (2.2%), highlighting the significant role of hypercoagulability in the pathogenesis of ACS in young patients with limited atherosclerotic lesions [52,56].

In a large Italian cohort of patients who developed acute MI at a young age, prothrombin G20210A showed a weak association with the premature onset of acute coronary thrombotic events. The prevalence of prothrombin G20210A was only 1.28 times that of controls [31]. Although the controls were individually matched to cases in terms of age, sex, educational level, and geographic origin, the results of this study may be biased by its case–control design. However, a retrospective Greek study enrolling patients with a history of STEMI before the age of 36 years found that prothrombin G20210A increased the risk of MI by 2.24 times [57]. After adjusting for traditional cardiovascular risk factors (hypertension, hypercholesterolemia, diabetes, and smoking), the risk further increased to 2.57 times.

It is important to emphasize that when the prothrombotic trait and classic cardiovascular risk factors coexist, the risk of MI increases significantly. Prothrombin G20210A carriers and either hypertensive, hypercholesterolemic, diabetic, or obese have five times increased risk of MI than non-carriers without metabolic risk factors [57]. Strikingly enough, prothrombin G20210A carriers who smoke had 22 times increased risk of MI compared to non-carriers who are also non-smokers (95% CI 9.192–66.517). Another study found that in patients with dyslipidemia older than 45 years, prothrombin G20210A increases the risk of MI by 4.5 times [58]. A strong synergistic effect between prothrombin G20210A and traditional cardiovascular risk factors was identified in young women who had a first acute MI under the age of 45 [59]. Those carriers and smokers had 43.3 times (95% CI 6.7–281) increased risk of MI compared to non-carriers and non-smokers. Similarly, those carriers with metabolic risk factors had 33.8 times (95% CI 5.5–209) increased risk of MI compared to non-carriers without metabolic risk factors. This means that in those with cardiovascular risk factors, prothrombin G20210A further increased the risk of MI approximately 4.5 times if they were smokers and more than 6 times if at least one of hypertension, diabetes, hypercholesterolemia, or obesity was present.

In postmenopausal women, this thrombophilic trait doubles the risk of MI. This risk, however, is not further increased by estrogen therapy [60].

Prothrombin G20210A is the second most common genetic thrombophilic trait in MINOCA patients. Among 84 MINOCA patients, prothrombin G20210A was found in 4.8% of patients [36]. The number of STEMI and NSTEMI cases was similar in MINOCA patients (5% vs. 4.5%). In another study, the prevalence of prothrombin G20210A was 4.4 times higher in MINOCA patients than in patients with MI and obstructive coronary artery stenosis. When only patients under 50 were analyzed, the risk became 4.9 times higher [15].

While a Danish study reported that the presence of both FV Leiden and prothrombin G20210A did not statistically significantly increase the risk of MI [18], others confirmed that the risk of MI increases when the two thrombophilic traits are associated. Concomitant FV Leiden and prothrombin G20210A were identified in 22.6% of young patients with MI [61]. The risk of MI is 1.82 when only one mutation is present and increases to 16.018 in the presence of double heterozygosity [28]. Carriers of both FV Leiden and prothrombin G20210A seem particularly susceptible to acute MI. Although double thrombophilia carries a greater risk of arterial thrombosis than either thrombophilia taken separately (Table 1), the risk of arterial thrombotic events—including MI, ischemic stroke, transient ischemic attack, and peripheral arterial thrombotic event—of those with FV Leiden and prothrombin G20210A is still ten times lower than their risk of VTE [62].

### 3.2. The Risk of Recurrence of MI

While for FV Leiden, there are more data available, for prothrombin G20210A, the literature is scarce (Table 2). Burzotta et al. showed that prothrombin G20210A adversely affects the long-term prognosis after a first acute coronary syndrome in two subgroups of patients, namely those without traditional cardiovascular risk factors and those not treated by revascularization procedures [63]. The two-year risk of events, including death from cardiovascular causes or MI or unstable angina, was 5.64 times higher in those without metabolic risk factors and almost three times higher in those without revascularization.

Patients with established CAD or a history of MI/revascularization procedures typically have a higher atherosclerotic burden and vulnerable plaques, placing them at increased risk for subsequent acute coronary events. Among survivors of a first MI, prothrombin G20210A carriers had a 1.8 times increased risk of recurrence of a major cardiovascular event, namely non-fatal MI, unstable angina, revascularization procedure, or cardiovascular death compared with non-carriers in an average follow-up period of 11 years [43].

In CAD patients undergoing coronary artery bypass grafting (CABG), the preoperative incidence of totally occluded coronary arteries and ventricular aneurysms were higher in patients with both FV Leiden and prothrombin G20210A mutation than in non-carriers [64]. However, none of the thrombophilic traits influenced the postoperative outcome, including MI and stroke.

**Table 1:** Recent studies assessing the association between FV Leiden and/or prothrombin G20210A and myocardial infarction.

Author, Year	Country	Cases		FV Leiden Prevalence of the Mutation/OR for the Association with MI	Prothrombin G20210A Prevalence of the Mutation/OR for the Association with MI
		No.	Age (Mean)% Sex		
Angeline et al., 2005 [65]	India	52 MI	NR	Prevalence = 5.8% No association with MI	Prevalence = 0% No association with MI
Zee et al., 2006 [66]	USA	523 MI	58.3 y M	OR = 1.23 (95% CI 0.81–1.86)	OR = 1.05 (95% CI 0.65–1.72)
Hindorff et al., 2006 [60]	USA	273 MI	67.5 y F	NR	Prevalence = 2.8% OR = 2.2 (95% CI 1.1–4.7)
Mannuci et al., 2006 [67]	Italy	520 ACS 54.2% MI	67 y 72.1% M	Prevalence = 3.8% for heterozygotes *p* = 0.99 for association with ACS	Prevalence = 4% for heterozygotes *p* = 0.87 for association with ACS
Smith et al., 2007 [5]	USA	856 MI	65.3 y 57% F	OR = 1.0 (95% CI 0.7–1.5)	OR = 1.4 (95% CI 0.9–2.1)
Morgan et al., 2007 [68]	USA	811 ACS 73% MI	60.7 y—M 63.1 y—F 68% M	OR = 0.93 (95% CI 0.58–1.50)	OR = 0.92 (95% CI 0.52–1.64)
Celik et al., 2008 [69]	Turkey	129 MI	≤45 y	Prevalence = 7.8% No association with MI	Prevalence = 1.1% No association with MI
Settin et al., 2008 [24]	Egypt	44 MI	52.2 y 81.8% M	Prevalence = 43.0% OR = 4.45 (95% CI 2.17–9.13) for heterozygotes Prevalence = 9% OR = 8.19 (95% CI 1.91–35.21) for homozygotes	NR
Martinelli et al., 2008, [26]	Italy	489 CAD (307 MI)	60.3 y 83.6% M	Prevalence = 2.9% for heterozygotes Prevalence = 0.3% for homozygotes *p* = 0.725 for association with MI	Prevalence = 5.9% for heterozygotes *p* = 0.204 Prevalence = 0% for homozygotes
Pestana et al., 2009 [70]	Venezuela	175 MI	57.87 y 75.5% M	Prevalence = 2.29% for heterozygotes OR = 0.76 (95% CI 0.16–3.69) Prevalence = 0% for homozygotes	NR
Weischer et al., 2009 [71]	Denmark	720 MI ^a^ 1123 MI ^b^	≥20 y	NR	HR = 1.7 (95% CI 1.1–2.7) for heterozygotes in general population studies OR = 2 (95% CI 1–3.8) for heterozygotes in case–control studies
Mannucci et al., 2010 [31]	Italy	1880 MI	39.6 y 89% M	OR = 1.66 (95% CI 1.15–2.38)—all MI patients OR = 1.00 (95% CI 0.58–1.72)—normal cholesterol OR = 3.58 (95% CI 2.19–5.86)—hypercholesterolemia	OR = 1.28 (95% CI 0.91–1.79)
Forte et al., 2011 [28]	Italy	164 MI	67 y 89% M	Prevalence = 5.4% for heterozygotes Prevalence = 0% for homozygotes No association with MI	Prevalence = 9.3% for heterozygotes Prevalence = 0.7% for homozygotes OR = 4.00 (95% CI 1.51–10.56)—all carriers
Dogra et al., 2012 [72]	India	184 MI	36.4 y 96.2% M	Prevalence = 7.1% OR = 3.7 (95% CI 1.5–9.5)	NR
Himabindu et al., 2012 [73]	India	51 ACS 86% MI	≤50 y 86% M	Prevalence = 0%	NR
Tomaiuolo et al., 2012 [27]	Italy	955 MI and 698 MI	38.7 y 62% M 69.5 y 65% M	Prevalence = 8.4% in W Prevalence = 3.5% in M and Prevalence = 3.5% in W Prevalence = 2.5% in M	Prevalence = 9% in W Prevalence = 4.2% in M and Prevalence = 3.4% in W Prevalence = 2.8% in M
Ben Slama et al., 2012 [32]	Tunis	100 MI	46.92 y 84% M	Prevalence = 9% OR = 1.55 (95% CI 0.58–4.12) Prevalence = 0% for homozygotes	Prevalence = 3% OR = 1.21 (95% CI 0.22–5.94) Prevalence = 0% for homozygotes
Onrat et al., 2012 [58]	Turkey	140 MI	42.04 y 57.19 y 60.7% M	Prevalence = 0.20% *p* = 0.293 (MI vs. healty controls) *p* = 0.741 (MI ≤ 45 y vs. MI > 45 y) Prevalence = 0% for homozygotes	Prevalence = 14.29% *p* = 0.469 (MI vs. healty controls) *p* = 0.535 (MI ≤ 45 y vs. MI > 45 y) Prevalence = 0% for homozygotes
Gonchar et al., 2012	Belarus	175 MI	NR	Prevalence = 6.9% OR = 2.41 (95% CI 0.96–6.02) *p* = 0.05 for heterozygotes Prevalence = 0% for homozygotes	Prevalence = 2.3% OR = 2.08 (95% CI 0.46–9.42) *p* = 0.56 for heterozygotes Prevalence = 0% for homozygotes
Sode et al., 2013 [18]	Denmark	2708 MI	≥20 y	OR = 1.0 (95% CI 0.9–1.2) for heterozygous OR = 0.9 (95% CI 0.4–2.1) for homozygous	OR = 1.3 (95% CI 1.0–1.7) for heterozygous No event among homozygous
Vaccarino et al., 2013 [74]	Italy	60 MI	≤46 y 100% M	NR	OR = 1.26 (95% CI 0.35–4.46) for heterozygous OR = 1.09 (95% CI 0.10–12.21) for homozygous OR = 1.22 (95% CI 0.39–3.82) for all carriers
Ezzat et al., 2014 [75]	Egypt	30 MI	45.5 y 80% M	Prevalence = 26.7% for heterozygous Prevalence = 3.3% for homozygous *p* < 0.05 for association with MI	NR
Kaur et al., 2014 [76]	India	184 MI 166 MI	36.4 y 96.2% M 64.1 y 74.7% M	Prevalence = 7.1% Prevalence = 3% OR = 2.4 (95% CI 0.9–7.0)	NR
Alkhiary et al., 2015 [61]	Egypt	31 MI	34.16 y 90.3% M	Prevalence = 51.6% for heterozygous OR = 6.48 (95% CI 1.56–26.84) Prevalence = 3.2% for homozygous *p* = 0.469 for association with MI	Prevalence = 45.2% for heterozygous OR = 4.6 (95% CI 1.13–19.24) Prevalence = 0% for homozygous
Hmimech et al., 2016 [54]	Morocco	100 MI	58.6 y 70% M	NR	Prevalence = 59% for heterozygous OR = 32.73 (95% CI 15.11–69.71) *p* < 0.001 for association with MI Prevalence = 10% for homozygous OR = 115 (95% CI 1.75–7332) *p* = 0.003 for association with MI
Rallidis et al., 2017 [57]	Greece	255 MI	<36 y 87.8% M	Prevalence = 7.8% for heterozygous *p* = 0.512 for association with MI Prevalence = 0% for homozygous	Prevalence = 7.4% for heterozygous OR = 2.24 (95% CI 1.10–4.25) Prevalence = 0% for homozygous
Milgrom et al., 2017 [77]	USA	153 79% MI	≤45 y 73.8% M	Prevalence = 7% *p* = 0.88 for association with MI	Prevalence = 3% No association with MI
El-Fatah et al., 2018 [78]	Egypt	120 MI	57.5 y 51.7% M	NR	Prevalence = 3.3% for heterozygous OR = 4.67 (95% CI 0.24–88.30) *p* = 0.303 for association with MI Prevalence = 0% for homozygous
Mohammed et al., 2018 [79]	Iraq	56 ACS	<50 y 96.4% M	Prevalence = 1.8%	Prevalence = 1.8%
Amara et al., 2018 [45]	Tunis	200 51% MI	62.71 y 61.5% M	Prevalence = 17.6% OR = 0.92 (95% CI 0.42–1.99), *p* = 0.86 (CAD + MI vs. CAD-MI) OR = 4.23 (95% CI 1.29–13.8), *p* = 0.02 (CAD + MI vs. control)	NR
Stepien et al., 2019 [36]	Poland	84 MINOCA	45.5 y 60.7% M	Prevalence = 14.3%	Prevalence = 4.8%
Msalati et al., 2021 [80]	Libya	69 MI	53.70 y NR	Prevalence = 3.27% *p* = 0.5 for association with MI	Prevalence = 2.5% *p* = 0.36 for association with MI
Golestani et al., 2022 [81]	Iran	150 MI	51.78 y 51.3% M	OR = 5.17 (95% CI 0.59–44.8) for heterozygous, *p* = 0.13 for association with MI OR = 1.6 (95% CI 0.00) for homozygous, *p* = 0.99 for association with MI	NR

MI = myocardial infarction; ACS = acute coronary syndromes; CAD = coronary artery disease; M = male; F = female; y = years; OR = odds ratio; RR = relative risk; ^a^ = in the general population studies; ^b^ = in case–control studies; NR = not reported.

**Table 2:** Recent studies assessing the association between FV Leiden and/or prothrombin G20210A and myocardial infarction recurrence.

Author, Year	Country	Cases		Outcome, Follow-Up Duration (Mean)	FV Leiden in Patients with Recurrent Events	Prothrombin G20210A in Patients with Recurrent Events
		No.	Age (Mean) % Sex			
Mannuci et al., 2006 [67]	Italy	520 ACS 54.2% MI	67 y 72.1% M	MACE—death, MI, or target lesion revascularisation 22.2 months	Prevalence = 3.7% *p* = 0.8 for association with MACE	Prevalence = 3.7% *p* = 0.8 for association with MACE
Van der Krabben et al., 2008 [43]	Netherlands	542 MI	56 y M	MACE—MI, UA, PTCA, CABG surgery and death due to coronary events 11 years	RR = 1.0 (95% CI 0.6–1.6)	RR = 1.8 (95% CI 0.8–4.1)

MI = myocardial infarction; ACS = acute coronary syndromes; UA = unstable angina; PTCA = percutaneous transluminal coronary angiography; CABG = coronary artery bypass graft; MACE = major adverse cardiac events; M = male; F = female; y = years; RR = relative risk; NA = not assessed.

## 4. Discussion

The pathogenesis of acute MI is the result of a synergistic effect between atherogenic and thrombogenic risk factors. In the elderly, acute MI is almost always the result of the destabilization of an atheromatous plaque followed by subocclusive or occlusive thrombosis. These patients have advanced atherosclerosis and multiple cardiovascular risk factors, whose cumulative power to trigger an ACS far exceeds that of hereditary thrombophilic states. Neither FV Leiden [25,26,28] nor prothrombin G20210A [52,53,55], by themselves or together [18], were significantly associated with acute MI in old age.

In patients with angiographically documented advanced CAD, the risk of MI was not correlated either with the presence of FV Leiden or with prothrombin G20120A when each polymorphism was considered individually [26]. However, the risk of MI increased when several hemostatic gene polymorphisms were added. The prevalence of MI increased progressively from 38.5% in patients with less than 3 unfavorable alleles to 62.6% in patients with 3–7 unfavorable alleles and, finally, to 78.3% in patients with more than 7 unfavorable alleles. This highlights that FV Leiden and prothrombin G20210A are underpowered, and only an increased number of prothrombotic alleles may confer a significant risk of developing MI in the elderly.

Acute coronary events in young individuals are generally surprising, with a strong psychological and economic impact at multiple levels: personal, familial, and social. Early and accelerated atherosclerosis in an individual with multiple traditional cardiovascular risk factors is the most common substrate, but in the absence of traditional cardiovascular risk factors and significant coronary atherosclerosis at angiography, other risk factors must be present to trigger rare cases of acute MI.

The importance of genetic factors seems to be particularly relevant in younger individuals. An extensive genetic analysis of this population showed that 35 single nucleotide polymorphisms (SNPs) in 12 genes were nominally associated with MI, but each SNP had only a modest effect size [82]. It was emphasized that MI has many genetic determinants and that the simultaneous presence of several polymorphisms with modest effects on the hemostatic pathway, through their summative consequences, significantly increases the risk of MI [83]. In a short series of patients who underwent coronary artery bypass grafting for early-onset CAD (under the age of 35), the prevalence of FV Leiden was statistically significantly higher than in age- and sex-matched healthy controls (*p* < 0.05) [84]. Patients with both FV Leiden and prothrombin G20210A had a 5–6 times higher risk of CAD than double non-carriers [62,71]. 

A significant risk of MI was noted in young women as well, in whom FV Leiden increased the risk of acute MI by 2.4 times [30]. Two other conditions with prothrombotic potential should be investigated in young women with ACS: the use of oral contraceptives and the presence of antiphospholipid syndrome. Exogenous estrogens increase the risk of both venous and arterial thrombosis [85]. However, the absolute risk of MI remains low at 10.1 per 100,000 [86]. Furthermore, while taking oral contraceptives, women with FV Leiden or prothrombin G20210A have a risk of MI similar to non-carriers [87]. Of note, in postmenopausal women, estrogen replacement therapy increased the risk of MI only in prothrombin G20210A carriers who were also hypertensive (OR = 4.32; 95% CI 1.52–12.1) [88,89]. Antiphospholipid syndrome (APS) is a systemic autoimmune disease characterized by recurrent arterial and/or venous thrombosis and/or obstetric events in the presence of persistent antiphospholipid antibodies. Acute MI has a prevalence of 1.2% in patients with primary APS and 3.8% in those with systemic lupus erythematosus-APS. Acute MI usually occurs in the fourth decade of life, is more frequent in women than men, and may be the first manifestation of the disease [90]. The coronary arteries are usually normal or have minimal lesions at coronary angiography. Recurrent thrombotic episodes and stent/graft thrombosis are common. The coexistence of FV Leiden/prothrombin G20210A with APS was highlighted by several case reports [91,92] and one case–control study [93]. However, there are still insufficient data to establish whether they have an effect on the risk of MI in women with APS.

The relationship between thrombophilia and MI became stronger when traditional cardiovascular risk factors overlap, with the risk increase reaching 25–32 times [30]. The risk of premature CAD is increased by 50% in patients with FV Leiden compared to age- and sex-matched non-carrier controls, higher in women compared to men. In carriers of FV Leiden and at least one metabolic risk factor, the risk of premature CAD is 25 times higher than in non-carriers without metabolic risk factors [94]. The association with diabetes mellitus may be of particular interest due to the high prevalence of the disease in the population and the extensive vascular damage that characterizes it [95]. An increased frequency of FV Leiden in young MI survivors was also identified in smokers [14,30,91,96].

A modest association between FV Leiden and the risk of MI was highlighted in several meta-analyses. The lowest risks were 1.21 (95% CI, 0.99–1.49) [97] and 1.22 (95% CI, 1.10–1.35) [19], while the highest risks were 1.6 (95% CI, 1.30–1.98) [98], and 2.46 (95% CI, 1.35–4.50) [99]. The weak effect of individual gene polymorphisms on the risk of MI emphasizes the principle that ‘‘the higher the allele effect, the lower the allele frequency” [100]. It implies that genetic variants relatively frequent in the general population could have only a mild effect on MI occurrence.

However, some countries, such as the Mediterranean ones, reported a high prevalence of FV Leiden and an important association between the prevalence of this thrombophilic trait and the risk of MI at a young age [24,61,75]. A meta-analysis of 42 studies mainly originating from European countries showed that prothrombin G20210A is a low-penetrant risk factor for CAD, but when restricted only to studies with MI patients, a borderline statistically increased risk was found [101]. A significant association was identified only in Europeans, which suggests that ethnicity may be a criterion when establishing an indication for screening for thrombophilia.

Inherited thrombophilia that result in coronary thrombosis is highly present among patients with MINOCA and is mainly represented by APC resistance/FV Leiden observed in 12–14.3% of patients and protein C or S deficiencies observed in 3% of patients [34,36]. Two brothers, homozygous for FV Leiden and heterozygous for MTHFR C677T polymorphism, had acute MI at 29 and 26 years, respectively. In each case, coronary angiography revealed the presence of thrombosis and the absence of any atherosclerotic lesion [96]. In those younger than 50 years, FV Leiden and/or prothrombin G20210A were present in 19.5% of MINOCA patients compared to 5.5% in patients with MI and obstructive CAD [15]. A large analysis of 12 studies focusing on thrombophilia screening, which enrolled 834 patients, reported a cumulative prevalence of the thrombotic disorder of 11% [102].

Early reperfusion therapy in STEMI, either fibrinolysis or percutaneous coronary intervention (PCI), significantly reduced the incidence of left ventricular (LV) thrombosis. Nevertheless, approximately 5.5% of patients still develop this complication, especially those who have suffered extended anterior acute MI [103]. However, the available data shows that FV Leiden and prothrombin G20210A are not risk factors for LV thrombosis in patients with acute MI [104]. A study that specifically assessed this risk in FV Leiden carriers with anterior acute MI did not find an increased risk for this complication [105]. Therefore, the decision to use anticoagulants in this setting will be based on the recommendations of the guidelines for the general population [106,107].

While FV Leiden and prothrombin G20210A significantly increase the risk of venous thrombosis, their role in arterial thrombosis is generally modest. FV Leiden and prothrombin G20210A have low significance in unselected patients with a personal or family history of arterial thrombotic events, so FV Leiden and prothrombin G20210A testing should not be conducted in a routine manner [108]. However, there are some exceptions that justify screening for these two thrombophilic mutations in survivors of an acute MI: (a) the event occurred in individuals under the age of 45; (b) the absence of traditional cardiovascular risk factors such as hypertension, dyslipidemia, obesity, diabetes, and smoking; (c) MI in the absence of a flow-limiting artery lesion at coronary angiography (MINOCA); (d) family history of VTE at a young age; (e) family history of FV Leiden or prothrombin G20210A mutation; and (f) women smokers younger than 50 years [108,109,110]. It must be emphasized that genetic testing for FV Leiden and prothrombin G20210A mutations does not require interruption of the antithrombotic treatment.

The identification of one or both thrombophilic traits will have immediate and long-term implications for practice. First of all, optimal control of modifiable traditional cardiovascular risk factors needs to be implemented in order to reduce the risk of recurrent events. Secondly, customized antithrombotic therapy should be considered. Thirdly, genotyping and genetic counseling of all family members of affected cases should be recommended for proper prophylaxis.

According to available data, the initial therapeutic management of the acute coronary event, namely reperfusion therapy, is not different in FV Leiden or prothrombin G20210A carriers compared to the general population. Given the lower bleeding risk under DAPT conferred by FV Leiden, extended DAPT may be considered in these patients. Bleeding occurs during DAPT in only 5.5% of FV Leiden carriers compared to 7.4% of non-carriers, the difference being mainly driven by minor bleeding, while major bleeding is similar [49]. The association of FV Leiden with bleeding is constant among the three possible antiplatelet combinations within DAPT—aspirin and ticagrelor/prasugrel/clopidogrel [46,47,111]. Other antithrombotic strategies have not been specifically assessed in FV Leiden carriers. Of note, no antithrombotic strategies have been specifically studied in prothrombin G20210A carriers. Whenever there are no specific recommendations, current guidelines for the treatment of acute MI in the general population should be followed. DAPT consisting of aspirin and a potent P2Y12 receptor inhibitor (prasugrel or ticagrelor) is recommended for 12 months following PCI and stent implantation [106,107]. Furthermore, NSTEMI patients without increased risk of major or life-threatening bleeding and with high or moderately increased ischemic risk are candidates for extended DAPT (>12 months). Recently, a new strategy for their antithrombotic treatment has emerged, namely aspirin and low-dose rivaroxaban, an anticoagulant that directly inhibits FXa. This therapeutic approach is useful in the first 12 months after PCI in patients with high ischemic and low bleeding risk. Moreover, it is an option for extended antithrombotic treatment in patients without increased risk for major or life-threatening bleeding and with high or moderate thrombotic risk. High ischemic-risk STEMI patients treated by primary PCI and stent implantation may receive extended DAPT with aspirin and ticagrelor for up to 3 years in the absence of bleeding complications [106]. Furthermore, low-dose rivaroxaban may be added in patients with low bleeding risk who receive aspirin and clopidogrel.

New research directions emerge as technology develops and the complex mechanisms of myocardial ischemia are understood. FV Leiden and prothrombin G20210A carriers have a greater extent and severity of ischemia assessed by myocardial perfusion SPECT than non-carriers. Furthermore, these thrombophilic traits were independent predictors for moderately and severely abnormal Summed Stress Scores [112].

Our study has several limitations. Firstly, the studied literature is heterogeneous regarding the quality and the amount of data provided. On the one hand, it includes case reports of MI in young people. Such an event triggers extensive investigations toward the identification of a prothrombotic substrate, usually resulting in the identification of multiple (hereditary ± acquired) thrombophilic traits. On the other hand, there are studies that included patients with MI, and that investigated one or several thrombophilic traits in the enrolled population, thus providing only limited data on the individual prothrombotic load. Some prothrombotic conditions, such as APS, that may contribute to MI in young women have not been systematically investigated. Moreover, our review is based on case–control studies that are encumbered by selection bias. Furthermore, the available data mainly come from studies with a relatively small number of patients. Considering the low prevalence of mutations in the general population, the relationship between thrombophilia and myocardial infarction, depending on the heterozygous or homozygous character of the mutations, could not be adequately evaluated. Secondly, there is heterogeneity in reporting MI. Some studies cumulatively report events from all arterial territories, namely cerebral, coronary, and peripheral arteries, while others include, along with MI, unstable angina and revascularization treatment. Therefore, it is difficult to identify only patients with acute MI. Thirdly, CAD is a multifactorial disease, and a polygenic approach would be desirable.

## 5. Conclusions

Factor V Leiden and prothrombin G20210A screening in unselected patient populations with myocardial infarction is not justified. However, it should be implemented in select cases, such as acute coronary syndromes in young individuals and/or in the absence of traditional cardiovascular risk factors and/or in the absence of significant coronary artery stenosis at angiography. Moreover, FV Leiden identification could be useful for guiding long-term antithrombotic treatment after an acute coronary event.

## Data Availability

Data sharing not applicable.
